# The Commercial Determinants of Health and Evidence Synthesis (CODES): methodological guidance for systematic reviews and other evidence syntheses

**DOI:** 10.1186/s13643-023-02323-0

**Published:** 2023-09-14

**Authors:** Mark Petticrew, Rebecca E. Glover, Jimmy Volmink, Laurence Blanchard, Éadaoin Cott, Cécile Knai, Nason Maani, James Thomas, Alice Tompson, May C. I. van Schalkwyk, Vivian Welch

**Affiliations:** 1 Faculty of Public Health and Policy, LSHTM, London, WC1H 9SH UK; 2 Department of Global Health, Faculty of Medicine and Health Sciences, Stellenbosch University, Stellenbosch, South Africa; 3 Dept for Health, University of Bath, Bath, UK; 4 Global Health Policy Unit, School of Social and Political Science, University of Edinburgh, Edinburgh, EH8 9LD UK; 5 UCL Institute of Education, University College London, 20 Bedford Way, London, WC1H 0AL UK; 6 Bruyère Research Institute, University of Ottawa, Ottawa, Canada

**Keywords:** Systematic reviews, Methods, Commercial determinants of health, Funding bias

## Abstract

**Background:**

The field of the commercial determinants of health (CDOH) refers to the commercial products, pathways and practices that may affect health. The field is growing rapidly, as evidenced by the WHO programme on the economic and commercial determinants of health and a rise in researcher and funder interest. Systematic reviews (SRs) and evidence synthesis more generally will be crucial tools in the evolution of CDOH as a field. Such reviews can draw on existing methodological guidance, though there are areas where existing methods are likely to differ, and there is no overarching guidance on the conduct of CDOH-focussed systematic reviews, or guidance on the specific methodological and conceptual challenges.

**Methods/results:**

CODES provides guidance on the conduct of systematic reviews focussed on CDOH, from shaping the review question with input from stakeholders, to disseminating the review. Existing guidance was used to identify key stages and to provide a structure for the guidance. The writing group included experience in systematic reviews and other forms of evidence synthesis, and in equity and CDOH research (both primary research and systematic reviews).

**Conclusions:**

This guidance highlights the special methodological and other considerations for CDOH reviews, including equity considerations, and pointers to areas for future methodological and guideline development. It should contribute to the reliability and utility of CDOH reviews and help stimulate the production of reviews in this growing field.

## Background

The complex and often negative links between the commercial sector and health are increasingly referred to as the commercial determinants of health (CDOH). CDOH research is in rapid growth, given additional impetus by the establishment of the Economic and Commercial Determinants of Health programme at the World Health Organization (WHO) [1]. CDOH research has often focussed on products such as ultra-processed foods, tobacco, sugar-sweetened beverages and alcohol as drivers of non-communicable diseases such as type 2 diabetes, cancers, cardiovascular disease and obesity, as well as on analysing the activities of the fossil fuel, breastmilk substitutes and other industries. Potentially any industry or commercial product or actor (such as an industry, or an individual or organisation related to or acting—transparently or not—as a front group or representative for an industry) comes within the remit of CDOH [2, 3] and recent CDOH analyses have focussed on the gambling, firearms, social media and pesticide industries, among others [4–9].

Action on CDOH requires evidence on effective solutions, and, as in other fields, key tools to help achieve these goals are evidence syntheses (systematic reviews, and related types of review). These include systematic reviews of the effectiveness of interventions (e.g. evaluations of policies and other interventions to reduce the impact of harmful commodities and to address industry strategies) [10, 11] and systematic reviews exploring the impact of commercial drivers of health, such as advertising and marketing [12, 13]. They also include reviews focussed on the acceptability of interventions, and research on the processes and mechanisms by which CDOH-related interventions work. ‘Interventions’ of interest to CDOH can thus include, not just health-promoting interventions, but also the strategies and other activities of harmful industries that they promote as ‘interventions’—for example, the educational activities of the alcohol, fossil fuel, tobacco, e-cigarette, pesticide, coal and other industries, as well as their lobbying activities, and other activities to distort science and misinform the public and policymakers [13–16].

CDOH interventions also include those which aim to curb the negative effects of commercial products, practices and influences. While much of CDOH research focusses on the harms of specific products, such as tobacco or alcohol (see definitions in Table 1), the Lancet Commission on the Commercial Determinants of Health has emphasised that even products that are often seen as benign can also cause avoidable health and social harms, giving as examples the financial sector’s role in the so-called deaths of despair, social media’s harmful effect on mental health [17] and the pharmaceutical industry’s use of intellectual property protections to secure high prices, and restricting access to essential drugs, despite massive public investment in their development [18]. It also notes that it is the practices and not just the products of major commercial entities that can harm health and widen inequities. The Lancet Commission on CDOH notes that current definitions often overlook the diverse effects of the commercial sector, and thus proposes a broad definition of the commercial determinants of health as: “the systems, practices, and pathways through which commercial actors drive health and equity” [18].

**Table 1:** Commercial determinants of health: definitions

WHO definition of commercial determinants of health [1] • *“Commercial determinants of health are the private sector activities that affect people’s health positively or negatively.* • *The private sector influences the social, physical and cultural environments through business actions and societal engagements; for example, supply chains, labour conditions, product design and packaging, research funding, lobbying, preference shaping and others.* • *Commercial determinants of health impact a wide range of health outcomes including obesity, diabetes, cardiovascular health, cancer, road traffic injuries, mental health and malaria.”*
West and Marteau (2013): “Factors that influence health which stem from the profit motive” [19, 20].
Kickbusch et al. (2016): “Strategies and approaches used by the private sector to promote products and choices that are detrimental to health” [21].
Kosinska and Ostlin (2016): “A good or a service where there is an inherent tension between the commercial and the public health objective”, including where the public health imperative is to reduce use or consumption and the commercial imperative is to increase this, or, conversely, where the public health objective is to increase accessibility and affordability and the commercial objective is to reduce this [4, 22].
Lancet Commission on the Commercial Determinants of Health (2023): “The systems, practices and pathways through which commercial actors drive health and equity” [18]

### What is CDOH research?

The interventions to restrict such harms are wide-ranging and include international policies and frameworks (like the Framework Convention on Tobacco Control) as well as interventions aimed at individuals (such as counter-marketing interventions [23]). The primary research which feeds into CDOH-focussed systematic reviews is similarly highly heterogeneous. It potentially includes basic research on mechanisms (e.g. research on the means and pathways through which commercial determinants influence health); research on prevalence (e.g. on the nature, size and impact of problems including research on the impact of harmful commodities); process evaluations; economic evaluations and other economic research; and modelling studies.

In this guidance, we adopt a definition of CDOH research which involves not simply research on products, but research which also includes the analysis of harmful (and other) industries’ behaviours, strategies, and tactics, as well as the environments they create which influence health directly and indirectly.

Of course, many aspects of the existing review guidance [24–27] will also be applicable to reviews with a CDOH focus. However, CDOH research poses its own particular challenges, and the blanket application of existing guidance may not always be appropriate. It may even be harmful, resulting in reviews which reach misleading conclusions. Take, for example, sponsorship bias, where the funding source of research can influence the type of research that is conducted and its outcomes. This is well-documented in pharmaceutical research and has also been documented in research sponsored by the tobacco and e-cigarettes, alcohol and food industries [28–34], where industry-funded research often preferentially focusses on particular research questions which aim to deflect attention from the industry or commodity in question.

Financial and other conflicts of interest (COIs) can significantly affect review quality. A Cochrane review has reviewed the evidence of financial conflicts of interest in systematic reviews and their associations with results, conclusions and methodological quality and found that in reviews of drug and device studies, systematic reviews with financial conflicts of interest more often have conclusions that are favourable to the funder and tend to be of lower methodological quality [35].

Similarly, Barnes and Bero have found that tobacco industry-funded reviews are about 90 times as likely as reviews funded by any other source to conclude that passive smoking is not harmful [36]. Sponsorship of review articles by the tobacco industry also influenced the conclusions of these articles independent of methodological quality. In the case of food industry funding, it has been found that studies are 21 times more likely to report unfavourable results about nutrition labelling if the authors declare a conflict of interest or if the study is funded by the food industry [37].

The non-publication of primary studies, particularly studies with findings that are unfavourable to the funder, is also an ethical concern; non-publication of reviews is also common, and 44% of meta-analyses in the pharmaceutical industry go unpublished [38].

One implication for evidence synthesis is that in order to produce adequate, non-misleading evidence to protect population health, reviewers must consider not just the *methods* of the primary studies, but also their *framings*, and consider not just what is reported in the studies, but what is missing, unstated or misrepresented (for example, which questions, analyses and/or findings are selectively omitted and/or emphasised). This could form part of the critical appraisal stage of the review. Such analyses can be informed by the growing theoretical and conceptual literature on CDOH, and in particular by the literature on how corporations create and use industry-friendly evidence [13, 15, 18, 39–42].

### Equity considerations in systematic reviews on CDOH

CDOH as a field has a strong equity focus, because the exposure, consumption and harms of harmful commodities are strongly socially patterned; the marketing of harmful commodities is also commonly targeted at poorer communities and is a driver of other inequalities (see also Reporting the review: includes contribution of authors, declarations of interest, sources of support, ethical approval section). The most common non-communicable diseases (NCDs) that are responsible for 70% of deaths globally are driven by four main risk factors: tobacco use, physical inactivity, the harmful use of alcohol, and unhealthy diets; all of these factors are strongly commercially influenced, and almost three quarters of all NCD deaths occur in low- and middle-income countries (https://www.who.int/health-topics/noncommunicable-diseases#tab=tab_1). The Cochrane/Campbell Equity Methods Group guidance notes that one important aspect of equity relates to reviews and research in low- and middle-income countries (LMICs) (see: https://training.cochrane.org/handbook/archive/v6.2/chapter-16).

Alcohol, gambling and tobacco marketing and consumption, and the political and commercial activities of the industries behind the commodities, are drivers of gender, age, socioeconomic and other inequalities in health [39, 43–45]. Of course the same can be said of other industries, particularly the health care industry, which employs many of the strategies of harmful commodity industries, including hiding and misrepresenting the harms of products, misleading marketing, manipulating and distorting evidence, as well as healthcare-specific activities such as disease-mongering—that is, expanding the boundaries of treatable or apparently treatable disorders in order to increase sales of commercial products, such as pharmaceuticals [46].

Commercial actors often specifically target poorer communities, or individuals with protected characteristics. For example, the tobacco industry targets black communities with its products; the alcohol industry targets women, children, young people and LBGTQ+ people, and many harmful commodity industries target financially poorer users [47–49]. The PRISMA Equity extension may therefore be of relevance when reporting CDOH reviews [50].

Mission or policy capture by industry can also be a significant driver of inequities [51]. CDOH reviews therefore need to routinely incorporate an equity perspective. However, while current equity tools are useful for research relevant to CDOH, there are some key areas of divergence (including participation in research) that mean that CDOH systematic reviews require additional explicit guidance that builds on existing equity guidance, but also extends this to explicitly consider the conditions of research where the profit motive runs directly or indirectly counter to the values of public health.

### Aims of this guidance

The CODES guidance aims to identify the main areas where existing systematic review practice and methodological guidance may converge and diverge, and to propose potential solutions. For the purposes of this guidance, we define “evidence synthesis” to include a wide range of types of review including both narrative systematic reviews and meta-analyses, as well as scoping, mapping and other reviews. It also includes reviews that combine the synthesis of research studies with primary research data. In addition, the guidance considers reviews of both qualitative and quantitative research, with any type of research question or aim (i.e. not just reviews of effectiveness).

## Methods

To develop the guidance an informal consensus-based approach was taken, in which the main sources of review guidance were reviewed by the authors, and from these documents, the key steps in systematic reviews were identified. For each step, the CODES authors considered what the main implications of a commercial determinants perspective are for systematic review methods; for example, whether doing a systematic review on commercial factors has particular implications for the search strategy, and whether this might affect the conclusions of the review. To help this process, we identified CODES team members with experience across a number of dimensions: experience in a range of review methods (including reviewing and integrating qualitative and quantitative evidence, and reviews with a complexity focus); experience in reviewing evidence with a particular focus on LMIC settings; experience in equity aspects of systematic reviews (hence the collaboration with the Campbell/Cochrane Equity Methods Group); and specific experience in conducting CDOH reviews and primary research. We also sought to include members with experience in conducting reviews of health sector and pharmaceutical interventions, to broaden the field of expertise beyond the typical focus of CDOH research on harmful products (e.g. tobacco, alcohol, ultra-processed foods, etc.).

For the guidance, we have taken as an initial conceptual/methodological framework the Cochrane MECIR (Methodological Expectations of Cochrane Intervention Reviews) standards [52, 53]. This framework sets out expectations for the general methodological approach to be followed in a systematic review. As noted above, not all CDOH reviews will be reviews of interventions, so we have referred where necessary to other widely used guidance; however, for reasons of clarity and length, our main focus in this paper is on reviews of interventions.

The main additional sources of guidance are as follows: (i) guidance on reviews of qualitative research—see Cochrane Qualitative and Implementation Group (https://methods.cochrane.org/qi/); (ii) guidance for reviews of risk factor epidemiology (COSMOS-E) [54]; (iii) guidance on systematic reviews with a complex systems focus [27]; (iv) the PRISMA 2020 statement [55]; and (v) equity guidance from the Campbell and Cochrane Equity Methods Group, which develops and disseminates methods of incorporating an equity perspective in systematic reviews [50]. Where the review includes synthesis of qualitative CDOH research, the ENTREQ guidance will also be relevant [56].

## Results: key considerations at the main stages in a CDOH-focussed systematic review

This section presents the key stages in a systematic review, setting out how each might be interpreted or reinterpreted from a CDOH perspective. It then identifies potential methodological or other implications and the decisions that reviewers may need to take. (The MECIR stages are noted where relevant.)

### Involving stakeholders, co-production, Public Patient Involvement (PPI) [MECIR stage]

The Cochrane Handbook advises that:

*“Given the opportunity cost involved in undertaking an activity as demanding as a systematic review, authors should ensure that their work is relevant by: (i) involving relevant stakeholders in defining its focus and the questions it will address”.*

For CDOH reviews, a key challenge is that stakeholders may have significant conflicts of interest. Industry may be a stakeholder, but it will generally be inappropriate and unethical to involve, say, the food or alcohol industry in shaping the science in this way. Co-production of public health research in this sort of partnership with industry stakeholders is risky, and the alcohol, tobacco and other industries have previously demanded the right to influence and interpret research, and to set the parameters for such research [57, 58]. Involving stakeholders may undermine the credibility of the review and the researchers and result in biased science [28, 59]. As Lundh et al. showed, funding conflicts of interest (COIs) are also associated with lower-quality reviews [35]. Industry stakeholders will of course have the same opportunity as others to comment on the findings after the review is published.

The public is an important non-industry stakeholder, and Public and Patient Involvement (PPI) in reviews and research is in some cases a requirement of research funders. In the case of PPI, one particular challenge is posed by the capture of charities and patient representatives by industry, and such relationships are often undisclosed [60, 61]. A systematic review by Fabbri et al. examined pharmaceutical or medical device industry funding of patient groups and found that industry-funded groups tended to have positions favourable to the sponsor [62]. Reviewers therefore need to carefully manage PPI in CDOH reviews and it may be necessary to exclude those with significant COIs. However, Bero and Parker note that the lack of transparency around pharmaceutical industry support for consumer groups means that it is often difficult to know whether consumer voices have financial links to the manufacturers of the products they support [60].

### Developing the protocol of the review [MECIR]

Registering a review (e.g. on PROSPERO database [63]) and pre-publishing the protocol are seen as good practice. However, for sensitive CDOH topics where industries have a track record of attacking researchers and undermining the science, it may not be feasible to disseminate research plans in advance in this way. One solution may be to register the protocol with the funder or institution, but not to make it publicly available until the end of the project, or to register it with the Open Science Framework using their systematic review registration form (https://osf.io/by27q/), with the protocol available on request. However, registering or publishing a protocol is not in itself an indicator of the quality of the review, nor of a lack of bias. It is not difficult to publish a protocol, and predatory journals are happy to accept them from any source [64]. British American Tobacco has published a protocol for one of its studies in a predatory journal which has a dog (“Olivia Doll”) as an editorial board member (https://twitter.com/DrOliviaDoll1/status/1564413985344339968/photo/1).

### Setting the research question(s) to inform the scope of the review [MECIR]

The Cochrane Handbook advises that “Systematic reviews should address answerable questions and fill important gaps in knowledge” and that “The FINER criteria have been proposed as encapsulating the issues that should be addressed when developing research questions. These state that questions should be **F**easible, **I**nteresting, **N**ovel, **E**thical, and **R**elevant” [24].

One key consideration from a CDOH perspective is that not all review questions that are relevant, feasible or answerable are ethical. Industry may select questions for research and for reviews (or may select review questions to fund) which only meet its interests, rather than the public interest or scientific need. This is not to say that industries do not fund reviews which also answer legitimate or useful scientific questions, but there is evidence that industry funding can shape the review questions in ways that do not match public health priorities [65]. One obvious example is that the pharmaceutical industry funds medical interventions rather than preventive interventions, which would compete with its market, and promotes narratives which support industry-friendly market-driven solutions to public health problems (e.g. as in the case of antimicrobial resistance [66]). There are many other examples. The Coca-Cola company-funded research focussed on physical activity, to help distract from the role of sugar-sweetened beverages (SSBs) in obesity [67]. A recent scoping review on drinking in alcohol use amongst gender and sexual minorities, which was funded by the alcohol industry-funded charity Drinkaware, included review questions on prevalence, alcohol change during the life course, and interventions [68]. The obvious missing review question however is that about the drivers of alcohol use in such groups, where there is a growing literature on the role of the alcohol industry in marketing to LGBT+ communities [69].

Even without direct industry involvement in a review, systematic reviewers might inadvertently be complicit in advancing this commercial agenda—even if their review is conducted to the highest methodological standards. For example, most nutrition reviews (including Cochrane reviews) focus on the effects of single nutrients rather than dietary patterns; 50% of Cochrane nutrition reviews focus on nutritional supplements only, and 82% frame their questions using a medical paradigm, in which inadequate intake of single or multiple nutrients causes some deficiency or physiological dysfunction in individuals and populations, as opposed to a socio-ecological paradigm, which emphasises the wider social, physical and commercial determinants of dietary problems—such as the widespread availability and marketing of ultraprocessed foods and other foods with a harmful effect on health [70]. As the primary studies are mostly funded by industry, this is an example of how the food industry can indirectly influence the choice of review topics—and thus influence the preferred “solutions” to diet-related poor health. Systematic reviews can therefore inadvertently promote individualistic, industry-friendly solutions even in high-quality reviews, if they do not take a wider view of why certain types of intervention are selected, funded, evaluated and published, and by whom, and why—as well as the opportunity costs of such interventions.

Methodological rigour alone in systematic reviewing is therefore not a defence against industry-related biases and industry-friendly problem framings.

The preponderance of reviews of biomedical interventions such as drugs, vaccines and therapeutics as opposed to social, economic and legal interventions with an impact on health probably occurs for similar reasons.

As noted above, CDOH research is highly heterogenous (like any field of public health research), and includes for example:

- Basic research on *mechanisms* (e.g. research on the means and pathways through which commercial determinants influence health, and research to help understand the dynamics of CDOH systems)
- Research on the effectiveness of interventions to address CDOH (e.g. evaluations of policies and other interventions to reduce the impact of harmful commodities like alcohol, gambling and tobacco)
- Some definitions include *research on prevalence* (e.g. on the nature, size and impact of problems including research on the impact of harmful commodities); however, many CDOH researchers would say that CDOH research always involves a clear link with the activities of industry/private sector actors (not just research on the effects of specific commodities).
- Process evaluations (e.g. studies of the acceptability and implementation to prevent or mitigate CDOH harms, and studies of the acceptability of CDOH-related interventions)
- Research on industry strategies and tactics (including Corporate Social Responsibility (CSR) campaigns [71] and other industry interventions)
- Research on effects of industry and industry funding on study outcomes, and on researchers themselves [28, 31]

### Designing an initial logic model [72]

A logic model is required by some funders as part of the application process; however, in CDOH reviews, a systems diagram may sometimes be more useful (though logic models and systems diagrams often share many similarities) [73]. It may be particularly useful in CDOH reviews to consider the wider system-level influences on outcomes. For example, industry actors are not often considered as discrete parts of the causal chain in ‘traditional’ reviews, but in CDOH research they are conscious actors that can both intervene on, and can be subject to interventions (such as policies). Complex system diagrams may be helpful in CDOH reviews to capture such complexity. This can involve visualising the system with the help of causal loop diagrams [73] which may help with unpacking the individual system components and interconnections between them, and can help focus a CDOH systematic review’s research questions. They may also help focus the inclusion and exclusion criteria (e.g. by clarifying what evidence will be needed to elucidate a particular pathway within the diagram).

It should be noted the outcome of a CDOH analysis or review might itself be a logic model—one which shows the dynamics of the system in which the commercial determinants operate. For example, an ‘initial’ logic model may be developed by the review team (with input from appropriate stakeholders) and this might then be updated as the review progresses, and a final version produced reflecting the body of evidence from the review. Developing an initial logic model is also an excellent point in the review to clarify concepts across the team and can be a useful communication device to seek wider engagement.

At this stage, it may also be useful to consider using an existing CDOH conceptual framework to help consider the wider commercial, social and political environment within which the review question ‘sits’, such as the framework developed by the Lancet Commission on CDOH [18].

### Setting the eligibility criteria for including studies in the review [MECIR]

For reviews of complex interventions, it is common to include a wide range of study designs. This inclusiveness also makes sense for *non*-effectiveness reviews. CDOH reviews, too, might legitimately include a wide range of study designs and approaches. In particular, much CDOH research involves analysing documents and/or websites, though such studies do not feature in systematic review or critical appraisal guidance (i.e. how to review and incorporate different types of document analysis which use qualitative or quantitative methods). Nor do review methods for qualitative research map clearly onto evidence from studies involving document analysis. In CDOH reviews, reviewers may therefore need to consider how to integrate findings across very different types of study (and perhaps different research paradigms) within a single review. Existing review guidance may help with this, particularly guidance on integrating different types of evidence to help answer complex research questions [25, 74].

From an equity perspective, the effectiveness and functioning of CDOH interventions may differ between high-, middle- or low-income country settings and populations. There may also be differences in the wider context, e.g. economy and geography, and the relative importance of health issues. These considerations have implications for how review questions are framed and what studies are included, and the Equity Group guidance notes that it may be appropriate to include only studies conducted in LMICs when:

1. The intervention(s) that the review addresses is highly relevant in LMICs and of little or no relevance in high-income countries (HICs).
2. There are compelling reasons to believe that the problem or the intervention(s) are different in LMICs.
3. The outcomes of interest are different.
4. The intervention(s) would be expected to function differently, so that the evidence would be unlikely to be transferrable between LMICs and HICs.
5. The researchers or review commissioners are particularly interested in evidence from LMICs.

### Selecting outcomes (processes/mechanisms) to be addressed for studies included in the review [MECIR]

Identifying the appropriate primary and secondary outcomes is key for any effectiveness review. It is important to consider this selection process particularly carefully in CDOH reviews, because industry-funded studies often selectively include or emphasise particular outcomes, which may not be the ones of greatest health or policy importance. For example, tobacco, alcohol and other industry-funded educational interventions selectively focus on “knowledge” and “attitudes” as the main outcomes, while avoiding or under-reporting clinically meaningful outcomes such as changes in smoking, or alcohol consumption. Outcome switching may also take place, in which outcomes chosen post hoc make an intervention appear more effective. Commercial influences appear to have distorted the evidence on the effectiveness of remdesivir for COVID-19 for example [75]. Interim outcomes and preliminary findings from studies and preprints also require very careful handling as the selective publication or dissemination of positive findings may be an indicator of industry influence [76, 77].

A recent systematic review of trials comparing formula milk products in young children found evidence of selective outcome reporting between and within trials [78]. They concluded that some trials might have a marketing aim, and no robust scientific aim.

It is also important to consider whether there are other important data—not just health outcome data—which should be extracted from CDOH studies, which can help elucidate the wider causal chain or network. For example, CDOH research is interested in the extent to which commercial influences shape wider environments (e.g. marketing environments, and the media) which in turn affect consumption. Extracting and synthesising data on exposure to marketing, as well as data on behavioural and consumption outcomes, can help show how the wider commercial system influences health. This can help with supporting causal inferences from the evidence, as well as allowing the evidence from the review ‘speak’ to policymakers and the public.

Any review of interventions should extract data on unintended outcomes, including adverse effects. These adverse effects can include increased inequities [79, 80]. This is even more important in some CDOH reviews, particularly for interventions designed/implemented by industry, where potential adverse effects from a public health perspective may include increases in consumption, or where the interventions increase exposure to industry framings and narratives, even although the interventions are claimed to do the opposite. For example, alcohol industry educational materials in schools, which claim to be intended to prevent or delay drinking in schoolchildren, in fact appear to promote knowledge of drinking skills, and familiarisation with alcohol as a product [81, 82].

### Searching for studies (including publication bias) [MECIR]

In CDOH reviews, depending on the topic, searching may need to extend well beyond the common health databases; this is because CDOH research may also appear in business and related journals. For example, evidence on trade deals that lie on the causal pathway to NCDs, because they may increase the availability and/or affordability of unhealthy foods, may be found in the business or economic literature. Similarly, some modelling studies on the effects of alcohol, tobacco or other commodity pricing on consumption only appear in the economic and/or grey literature (e.g. EconLit, and the RePEc database http://repec.org/).

It is also common for industry evaluations not to be published in journals at all—studies carried out by market research companies may appear only as grey literature reports (e.g. company annual reports). Such documents may be published for their strategic and PR value rather than as evidence—so a full final report may never appear. Similarly, it is common for evaluations or other research commissioned by industry from academics never to be published in journals, but instead they may only be available directly from the industry organisation that commissioned the study, or from industry websites. As an example of this sort of industry ‘ghost evidence’, a review of Community Alcohol Partnerships found that evaluation evidence was often mentioned by industry bodies, and cited in presentations, but most could not be provided when asked for [83].

Publication bias may thus be a much greater risk in CDOH reviews. There is evidence that the tobacco industry has exploited the concept of publication bias to undermine the evidence on second-hand smoke [84]. Conversely, independent evidence may be missing because some journals are often risk-averse when it comes to publishing studies that are critical of industry; this also biases the evidence base. Non-publication of negative findings may also play a role, for example where studies of the ineffectiveness of industry-preferred interventions may remain unpublished.

Publication bias also appears to have significantly distorted the evidence on alcohol advertising; a review of the evidence found many advertising industry studies were published in advertising industry books that had never been cited in systematic reviews [85]. These studies showed that advertising works very effectively to influence consumption, contrary to the alcohol and advertising industry’s claims.

### Selecting studies to include in the review

In any review of the effectiveness of interventions to address CDOH, standard review methods will probably apply, and relevant evaluative studies may include quantitative (RCTs and non-RCTs) and/or qualitative studies. Reviews which include policy analyses, media analyses or legal evidence/witness testimony are more complicated; the primary studies may not fit within traditional study design taxonomies or evidence “hierarchies”, which in the health field are often epidemiologically based. As always, the key criteria for selecting and including studies in such reviews is to include the evidence which is the most appropriate for answering the review question, rather than trying to fit evidence into epistemological hierarchies which may not be suitable.

In some cases, evaluations or other studies may be mentioned (e.g. in annual reports), with claims about their effectiveness, but with too little accompanying information on the methods or findings to allow the validity of such claims to be judged. Reviewers may need to make a judgement about whether claims about outcomes without supporting methods really constitutes “evidence”, or not, and should therefore be excluded, or whether to include them, but—at the critical appraisal stage of the review—assess the strength or otherwise of the inferences that can be drawn from the information that is presented.

CDOH reviews often focus on reviewing harmful industry practices. Evidence on these sometimes comes from non-scientific sources, including whistle-blowers’ accounts, and investigative journalism. Methods for handling such materials are underdeveloped, but transparency in their selection and how they are integrated into an analysis is key, as for any review.

### Collecting data from included studies (including data extraction, description of studies, the PRISMA diagram, use of tables and diagrams)

Standard guidance on data extraction will generally apply to CDOH reviews. However, given that the influence of industry on science and on health-related decision-making is a key concern for CDOH research, it is essential to extract and include data on the authors’ affiliations, their COI declarations, and their/the primary study’s source of funding, and to include this in the review’s tables. Note also that when there is no declaration of COI or funding, this is not necessarily the same as “no COI”. COI or funding statements may be hidden in supplementary material, rather than in the main paper as required by most journals. Contacting authors for missing funding information should be routine in CDOH reviews. Note also that funding may be passed through front groups or industry-funded charities (‘charity-washing’) or organisations, and funding statements may not mention the specific industry which is the originating source (e.g. funding from Philip Morris’ Foundation for a Smoke Free World, where authors may report the funding, but not its tobacco industry source [86]). Industry funding may also be channelled through non-industry organisations or institutions, as with the alcohol industry’s funding of a controversial trial through the US National Institutes of Health (NIH). In this case, industry funding appears to have influenced the design of the trial to ensure it produced findings favourable to the funders [87].

It is also common for studies published by harmful commodity industries not to declare their funding, or to declare that it was funded by an industry-funded front group, without specifying which company or industry the funding originated from.

Checking funding may also require checking whether the authors’ organisations (i.e. not just the individual authors) have received industry funding. This is difficult to determine but can sometimes be assessed by examining institutional (e.g. academic) websites, and also the websites of funders themselves, though funders may not report which researchers or organisations they have funded. It can also involve checking authors’ declared funding in other published papers, other than those included in the review.

It will also be helpful to extract any information on the ethical aspects of studies, in particular whether ethical approval for the study was granted by any institution or organisation.

### Assessing the quality of evidence and summarising the findings [MECIR]

Generally, standard approaches to assessing the quality of the evidence will apply, though with the caveat that there may be many types of studies for which critical appraisal tools do not exist, or have been adapted for specific purposes and require validation– e.g. the critical appraisal of document analyses or media analyses. Commonly used critical appraisal tools also do not apply well to cross-sectional studies that do not involve humans (e.g. studies of commercial influences on living or working environments [88]). However, the issues they raise, such as selection bias and the identification of non-causal correlation, are relevant to CDOH reviews [88].

There is also a wider issue here which is worth considering, about how to handle the results of quality assessments of studies in systematic reviews. There is a long-standing tendency in systematic reviews to use the review simply as a way to identify methodological flaws in primary studies, before concluding that there is "no good evidence". (This has been described as ‘clever nihilism’ [89].) This overlooks the reality that no study is perfect, nor does it need to be, to be useful to decisionmakers. Taking critical appraisal to extremes can do harm if valid evidence is rejected [90]. The particular relevance for CDOH reviews comes from the fact that undermining the value of public health and other evidence is a key part of the harmful industry “playbook”, particularly where industries demand perfect evidence as a way to delay and defeat policies to protect health [91]. Ulucanlar and colleagues have shown this in a review of tobacco industry submissions to the UK Government Consultation on plain packaging for tobacco products. The tobacco companies undermined the evidence by employing their own experts to review published studies for methodological rigour in ways that did not conform to normal scientific critique approaches (an approach which has been called ‘mimicked scientific critique’ [42]).

The takeaway message for CDOH reviewers is that there is a need to be careful and accurate about the evidence, and for reviewers to consider carefully what are the best study designs that can be used for a specific research question, rather than focussing on what is ideal, but not feasible. Simply rating all evidence as “low” or “very low”, for instance because there are no RCTs when these are not feasible, risks sending the misleading message to policymakers or others that there is little evidence and that they may as well use people’s opinions instead. The history of tobacco (and indeed the gambling, alcohol and other industries) is one of denying that the evidence is ever “good enough” to act upon. While assessing study quality is important in a review, the purpose of a review is not simply to synthesise evidence, but to assemble evidence with the aim of supporting decision-making. Public health policymakers will in any case often act in accordance with the precautionary principle, in which lack of scientific certainty must not be used as a reason to ignore or postpone action to protect health [92].

In practice, this means that CDOH reviews in particular need to consider carefully whether the weight of evidence that is being reviewed presents “good enough” evidence of harms, or effectiveness (depending on the focus of the review), rather than simply treating the review as a methodological commentary on the weaknesses of the evidence. High methodological quality of the primary evidence on its own is not an assurance of the quality of the review; a systematic review which includes RCTs of narrowly defined, industry-friendly, contextless questions may be more inappropriate than a review which includes complex primary studies of a wide range of designs relevant to the review question. Thus, a transparent consideration of the ‘fitness for purpose’ of the evidence, as much as its methodological quality, will be of value. See Gough [93] and Gough et al. [94] for further details of such approaches, and a description of a weight of evidence framework for integrating evidence [25].

### Synthesising the results of included studies (includes assessing heterogeneity, subgroup analysis, sensitivity analysis, and investigating risk of bias due to missing results)

#### Narrative synthesis

In general, systematic reviews which adopt a narrative approach to synthesis may be more common in CDOH than reviews which synthesise effect sizes. An example of the former is the review by Savell et al. which had as an aim “To systematically review, using a qualitative, narrative synthesis approach, papers examining alcohol industry efforts to influence alcohol marketing policy, and compare with those used by the tobacco industry” [95]. The review identified five main political strategies and five main frames from seventeen studies. Another example is a review of tactics used by harmful industries to generate doubt. It reviewed, amongst other sources of evidence, scholarly books, peer-reviewed articles, well-researched journalism pieces and legal evidence related to five disparate industries and organisations selected for their destructive impacts on environmental and public health (tobacco, coal, and sugar industries, manufacturers of the pesticide Atrazine, and the Marshall Institute, an institute focused on climate change research) [96].

As noted above, doing research on industry strategies often involves qualitative methods, so CDOH systematic reviews might usefully draw on guidance on how to systematically review qualitative research, for example where the synthesis involves drawing together high-order themes across studies [97]. However, this may not be adequate for reviewing studies which involve discourse analysis, as the review may need to go beyond simply drawing out themes. For example, they may also need to consider synthesising evidence on contexts, framings and power relationships [98].

#### Meta-analysis and subgroup analysis

Meta-analysis may be possible where there are sufficiently similar studies with effect size data that have been reported (or can be treated) in a way that allows statistical pooling, as with any other review. In this case (and for narrative CDOH reviews too) subgroup analysis should be considered, such as analysing subgroups of studies stratified by source of funding. It is known that industry-funded studies in many fields over-report effectiveness and are systematically different in other ways. For example, systematic reviews undertaken by authors with histories of alcohol industry funding are more likely to study broader outcomes, to be cited more widely, and to exclusively report favourable conclusions [31]. The influence of food, soda, tobacco, gambling and pharma industry funding on the choice of research questions—typically reframing the research agenda towards industry-friendly areas—and on study outcomes is also well documented [7, 28, 59, 99–101]. Subgroup analyses which group studies by COIs, funding, and/or authors’ affiliations may therefore be useful to explore these issues. Of course, COI statements may be missing; COI disclosures are less frequently included in systematic reviews of Health Policy and Systems research compared to systematic reviews in other fields [102].

### Reporting the review: includes contribution of authors, declarations of interest, sources of support, ethical approval

Standard reporting criteria for reviews also apply to CDOH reviews (e.g. the PRISMA guidelines, and PRISMA extensions), and the PRISMA Equity extension may be of value [50]. Where the review includes synthesis of qualitative CDOH research, the ENTREQ guidance will be relevant [56].

The problem of COI’s in primary studies is mentioned above, and COIs of the review team and financial and other sources of support should be reported. A particular consideration for CDOH reviews is that industry sources have often tried to present scientific expertise as a form of bias—where expert knowledge of a topic is claimed to result in over-stating of study findings, so-called ‘white hat bias’ [103]. This is considerably over-stated; while intellectual COIs do exist, financial COIs are more impactful and a greater ethical priority [104].

Any effect of such bias is moreover overwhelmed by the effects of industry bias. A 2018 systematic review of the effect of financial conflicts of interest involving the food industry on nutrition studies found that industry-related studies were overwhelmingly more likely to reach ‘weak/null’ conclusions compared with independent studies regarding the adverse effects of SSB consumption on health—with an OR of 57·30 [105].

It is worth noting also that the Declaration of COIs does not remove bias, and declaring a COI may make the reader *over-*estimate the reliability and trustworthiness of the study [106]. A more effective solution is to prevent the funding or COI bias in the first place [107].

### Planning the update of the review

CDOH interventions may be independent of industry or may be industry-funded and/or delivered. In any review of industry-funded interventions, the interventions may change rapidly; interventions (e.g. public-facing education campaigns) may be withdrawn suddenly because of criticism; industry priorities may shift; and industry claims about the importance or effectiveness of those interventions may change. On the other hand, harmful commodity industries might continue with initiatives even when there is little or no independent evidence that they work [108, 109]. Yet industry may claim that there is a significant and growing evidence base supporting their use (alcohol industry school-based education programmes [82] and safe storage of firearms and pesticides are just some examples) [83, 110–112]. One implication of this for updating reviews is that we often assume that we only need to update a systematic review when the evidence base changes. However, in CDOH reviews, where the evidence is contested and subject to “spin”, it can be important to update a review even when there has been no apparent change in the evidence, in order to fully test industry claims.

## Discussion

CDOH is a field which is still in development, and this guidance is likely to change over time. However, it offers initial pointers to areas where current systematic review and evidence synthesis practice may diverge or may be nuanced differently. We believe that a failure to adapt existing review practice to take account of the nature of CDOH research may result in biased reviews, and result in conclusions which are misleadingly and inadvertently favourable to harmful industries; which in turn poses risks to health. The methodological developments necessary to address CDOH in evidence synthesis mirror those necessary in other areas, such as intervention complexity, and emerging guidance from ‘adjacent’ areas may be helpful for CDOH too, such as guidance on addressing complexity and on integrating different types of evidence [27, 74, 113, 114].

The guidance has focused on the main aspects of the conduct and reporting of methodological aspects of the review. There are other stages of a review’s conduct and dissemination which will benefit from applying a CDOH lens but are beyond the scope of this paper; for example, the dissemination of reviews, and non-academic dissemination activities, and the choice of journals to publish in, and whether to submit reviews to journals with industry-funded editors and editorial boards. “Publishing Addiction Science” by Babor et al. [115] is a valuable resource on publishing CDOH research, including systematic reviews. It has an applicability beyond the topic of addiction and includes advice on the challenges faced by researchers from developing and non-English-speaking countries [115].

The guidance has also encouraged a focus on equity; while it is difficult to capture all such dimensions of CDOH in a review, being aware of the wider political and social contexts within which interventions are implemented is important. At the level of the review itself, the PRISMA-Equity (PRISMA-E) extension to the PRISMA guidance, originally published in 2012 and updated in 2015, which seeks to improve the evidence base on how policies, resource allocation, and resource use can impact inequities, will be of use [50]. Additional research methods have recently been developed to ensure that inequities are not created or exacerbated by the research teams themselves [116]. These research tools and frameworks formalise and foreground concerns of equity during the systematic review process.

The PROGRESS-plus acronym can also help with assessing the differential health effects of interventions and incorporating an equity perspective into CDOH reviews, where the acronym stands for Place of residence, Race/ethnicity/culture/language, Occupation, Gender or sex, Religion, Education, Socioeconomic status, and Social capital. “Plus” stands for other factors associated with discrimination, exclusion, marginalisation or vulnerability such as personal characteristics (e.g. age, disability), relationships that limit opportunities for health (e.g. children in a household with parents who smoke) or environmental situations which provide limited control of opportunities for health (e.g. school food environment) [117].

The CODES guidance does not discuss the possible negative reception of some reviews. Where review findings do not accord with industry narratives, they may receive significant industry pushback in different forms. There is a separate literature on dealing with industry pushback, but dealing with it can include forewarning funders, policy colleagues and senior staff in organisations (e.g. Deans, Director, and comms teams), as well as seeking support and advice from other CDOH researchers and non-governmental organisations (NGO)s, who may have dealt with this before. Depending on the topic, it may also be necessary to send the review for legal review before publication.

Finally, implicit in this guidance is that the challenges of CDOH reviews are not just methodological. Doing such reviews may require us to rethink what harm is, what an intervention is, how to problematise what is often taken for granted, and what is worthy of study. Adopting a CDOH lens may require challenging our own assumptions and training about systematic reviews.

## Conclusions and next steps

There is a rich agenda for future CDOH research in relation to evidence synthesis, including methodological research. The latter might include reviewing the methods currently used in CDOH reviews. There is also further work to be done to incorporate an equity perspective within CDOH systematic reviews, and, conversely, to incorporate a more consistent CDOH perspective within reviews focussed on health equity. The integration of CDOH more fully into social determinants frameworks, where commercial determinants are often invisible, will in turn lead to more relevant reviews [118].

In conclusion, we hope that this guidance and future iterations of it will not just help fill evidence gaps, but will contribute to meaningful knowledge translation, that is, that it will stimulate the production of meaningful evidence to support decision-making by policymakers, practitioners, research funders and the public and their representatives.

## Data Availability

All relevant data/materials are available from the authors.
